# The Nigerian national blindness and visual impairment survey: Rationale, objectives and detailed methodology

**DOI:** 10.1186/1471-2415-8-17

**Published:** 2008-09-22

**Authors:** Brendan Dineen, Clare E Gilbert, Mansur Rabiu, Fatima Kyari, Abdull M Mahdi, Tafida Abubakar, Christian C Ezelum, Entekume Gabriel, Elizabeth Elhassan, Adenike Abiose, Hannah Faal, Jonathan Y Jiya, Chinenyem P Ozemela, Pak Sang Lee, Murthy VS Gudlavalleti

**Affiliations:** 1 International Centre for Eye Health, London School for Hygiene and Tropical Medicine, UK; 2 National Eye Centre Kaduna, Nigeria; 3 Specialist Hospital, Bauchi, Nigeria; 4 Ministry of Health, Dutse, Jigawa State Nigeria; 5 Ministry of Health Awka, Anambra State, Nigeria; 6 Vision Health Services, Ikeja, Lagos State, Nigeria; 7 Sight Savers, Country Office, Kaduna, Nigeria; 8 International Agency for Prevention of Blindness, Africa region, Ibadan, Nigeria; 9 Sight Savers West Africa Regional Office, Accra, Ghana; 10 National Programme for the Prevention Blindness, Federal Ministry of Health, Abuja, Nigeria; 11 Institute of Ophthalmology, UCL, London

## Abstract

**Background:**

Despite having the largest population in Africa, Nigeria has no accurate population based data to plan and evaluate eye care services. A national survey was undertaken to estimate the prevalence and determine the major causes of blindness and low vision. This paper presents the detailed methodology used during the survey.

**Methods:**

A nationally representative sample of persons aged 40 years and above was selected. Children aged 10–15 years and individuals aged <10 or 16–39 years with visual impairment were also included if they lived in households with an eligible adult. All participants had their height, weight, and blood pressure measured followed by assessment of presenting visual acuity, refractokeratomery, A-scan ultrasonography, visual fields and best corrected visual acuity. Anterior and posterior segments of each eye were examined with a torch and direct ophthalmoscope. Participants with visual acuity of < = 6/12 in one or both eyes underwent detailed examination including applanation tonometry, dilated slit lamp biomicroscopy, lens grading and fundus photography. All those who had undergone cataract surgery were refracted and best corrected vision recorded. Causes of visual impairment by eye and for the individual were determined using a clinical algorithm recommended by the World Health Organization. In addition, 1 in 7 adults also underwent a complete work up as described for those with vision < = 6/12 for constructing a normative data base for Nigerians.

**Discussion:**

The field work for the study was completed in 30 months over the period 2005–2007 and covered 305 clusters across the entire country. Concurrently persons 40+ years were examined to form a normative data base. Analysis of the data is currently underway.

**Conclusion:**

The methodology used was robust and adequate to provide estimates on the prevalence and causes of blindness in Nigeria. The survey would also provide information on barriers to accessing services, quality of life of visually impaired individuals and also provide normative data for Nigerian eyes.

## Background

In 2002 the World Health Organization (WHO) revised estimates of the global magnitude and causes of blindness which revealed a paucity of recent data for most countries in the African region [1]. Though Nigeria, is the most populated country in Africa, with a population of 135 million, no national data on the prevalence and causes of blindness exist [2]. Most data used for planning eye care services are generated either from urban areas where the large eye hospitals are situated [3] or from small, focal surveys [4-22]. These small studies indicate that blindness is likely to be a public health problem [4-22] but such data cannot be extrapolated to the entire country as the population is culturally, ethnically and geographically diverse. Similarly, national survey results from other West African countries (e.g. Benin, 1990; The Gambia, 1986, 1996, Cameroon, 1996) [23-26] may not be readily comparable to present day Nigeria, due to several years having passed since those studies were conducted. Differences in population size, demographic profiles, climate and eye care service accessibility and provision contribute in determining the frequency and distribution of conditions such as trachoma and onchocerciasis as well as other causes of visual loss (e.g. cataract, glaucoma).

**Figure 1:**
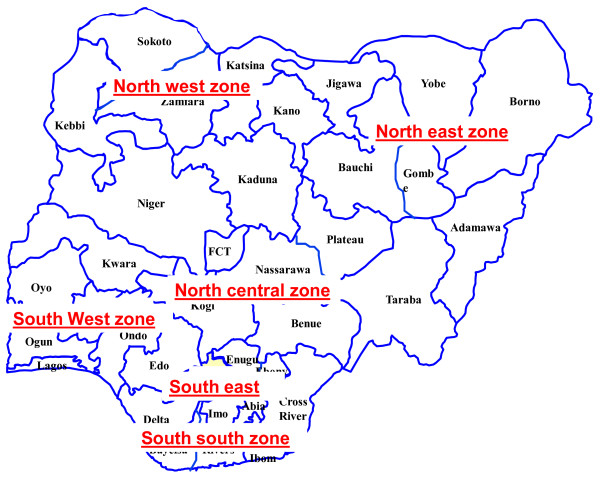
The Map of 36 states of Nigeria and the 6 geopolitical zones.

Nigeria is the ninth most populous county in the world and the most populated in Africa [27-29]. More than 500 languages are spoken in Nigeria which is home to more than 200 ethnic groups [2]. The population is projected to increase to nearly 210 million by the year 2025 [27]. The country is divided into 6 administrative zones (geo-political zones – GPZ), one Federal Capital Territory (FCT) of Abuja and 36 States [30] (Figure 1). Each State is subdivided into Local Government Authorities (LGAs), the smallest administrative division, of which there are 774 in the country [30].

Nigeria has five ecological zones (river delta, rain forest, transition, savannah and sahel) which are shared by 19 other countries with a total population of 345 million people in West and Central Africa. These ecological variations may have an important bearing on the prevalence and causes of blindness Life expectancy in 2007 is 46.8 years for males and 48.1 for females [31]. In Nigeria, 63% of the population lives in rural areas [28]. Adult literacy rate is 68% and the GDP per capita was 1,150 US$ in 2006 [31] with 70.2% living in poverty (<1 US$ per day) [2,29].

### Rationale and Objectives

Access to eye care services is especially limited in rural areas and amongst the urban poor. As such it is imperative that existent resources (human, financial, infrastructure and equipment) are used effectively, targeting the major avoidable causes of blindness in order that the goals of VISION2020 are achieved in Nigeria. The Nigeria National Program for Prevention of Blindness (NPPB) realises the importance of population-based data for evidence-based eye care planning in the country. Moreover, non-governmental organisations have planned to increase support to eye care activities for successful implementation of VISION2020 programmes in Nigeria, but can only do so if there is scientifically valid evidence on which to advocate, prioritise and plan.

The main objective of the survey was to determine the prevalence and causes of blindness and visual impairment among adults aged 40 years and older and children aged 10–15 years based upon a nationally representative sample. The other specific objectives were to:

1. Determine associations between climatic (ecological) zone, administrative area (geo political zone), place of usual residence (urban/rural) and other socio-demographic attributes (age, gender, social class, occupational categories, literacy levels etc.) and the prevalence and causes of visual impairment and blindness.

2. Identify priority regions and populations for blindness control

3. Assess the status of cataract surgical services by estimating cataract surgical coverage in association with socio-economic, demographic and geographic variables.

4. Determine visual outcomes after cataract surgery and identify factors associated with good (> 6/18) and poor (< 6/60) visual acuity.

5. Estimate the prevalence and describe types of refractive errors for the purposes of planning refractive error services (including those for children).

6. Generate normative data on parameters used in the diagnosis of glaucoma and for determining the range and distribution of the Intra Ocular Lens (IOL) powers needed for implantation after cataract removal.

7. Establish the magnitude and causes of functional low vision in order to assess the need for low vision services

8. Assess the impact of blindness and visual impairment on quality of life and visual functioning of affected individuals.

9. Identify barriers to the access of services by those visually impaired.

## Methods/Design

This paper describes the definitions, eligibility criteria, sample size calculation, enumeration procedures, visual acuity measurements, anthropometry and clinical examination procedures adopted for the study.

The age group 40 years and above was targeted based on the available evidence that most blindness occurs in this age category [32,33]. For example, in the Bangladesh and Pakistan national surveys the prevalence of blindness was very low among participants aged 30–39 years and increased exponentially after the age of 40 years [32,33]. Focussing on individuals aged 40 years and above also allows more accurate data on the causes to be collected, which is important in a country like Nigeria as it is thought to have more blinding eye diseases (e.g. Onchocerciasis) than countries in South Asia [32-40].

### Definitions Used for Identifying Appropriate Denominator

#### Household

A household was defined as all those living under the same roof and eating from a common cooking pot routinely. If the head of the household had more than one wife and the wife and children lived in a different compound, they were treated as a separate household.

#### Normal resident

Individuals who had lived in the cluster continuously for three months prior to the survey were labelled as a normal resident.

#### Eligible respondent

All individuals aged 40 years and above and residing continuously in the cluster for the preceding three months were eligible for inclusion as were children aged 10–15 years living in households which had an eligible adult. If the enumerators determined that a respondent was not going to be available over the following two days (when the survey team would be in the cluster), then the resident and his/her family were deemed ineligible.

#### Rural cluster

For the purpose of the survey, a rural area/cluster was defined as an inhabited village within an LGA with a population less than 20,000 (definition adopted by Nigerian Population Commission).

#### Urban cluster

Similarly, an urban cluster was defined as a settlement with a population of 20,000 or more. Smaller clusters adjacent to or located within large urban areas were also classified as urban if they had amenities similar to those found in large conurbations.

### Sampling design

The most recent census at the commencement of the survey was the 1991 census and the annual growth rate was estimated to be 2.9% [28]. The target population for the survey was extrapolated from the 1991 census using annual growth rates. The estimated target populations for each of the 6 GPZs ranged from 16 to 30 million in 2005 (Table 1). The proportion of the population aged 40 years and above was estimated to be 17.6% i.e. 23.6 million people in 2005 based on the 1991 census and the growth rate.

**Table 1:** Distribution of Nigeria population based on projections based on 1991 census.

**Geo political zone**	**Total population**	**% of total in each Geo Political Zone**	**Estimated population = > 40 years**	**% = > 40 years**	**Sample size = > 40 years**
North Central	18,312, 959	13.7	2,981,514	13.2	2,027
North East	22,211, 520	16.6	3,806,234	16.8	2,588
North West	30,120,187	22.5	5,147,360	22.8	3,499
South East	16,194,215	12.1	2,620,756	11.6	1,782
South South	20,221, 525	15.0	3,290,629	14.6	2,237
South West	26,237,689	19.6	4,657,051	20.6	3,166
Abuja Federal Capital Territory	558,829	0.4	112,335	0.5	76

**Total**	**133,856,924**	**100.00**	**22,615,879**	**100**	**15375**

Multi-stage stratified cluster random sampling; with probability proportional to size (PPS) procedures was used to identify a nationally representative sample of people aged 40 years and above. The sample was stratified by place of usual residence (urban/rural). Available demographic data indicated that 63.7% of the population overall lived in rural areas and 36.3% in urban areas [28]. However, the proportion of people living in rural areas varied by States and this was taken into account during stratification. In each GPZ and in the Federal Capital Territory (FCT) of Abuja, the proportion of clusters sampled was based on the proportion of the national population living in each of these administrative divisions. A sampling frame was constructed for each GPZ separately for urban and rural areas. Using a cluster size of 50 eligible adults, a total of 310 clusters were randomly selected across the country of which 226 (72.9%) were rural and 84 (27.1%) were urban.

In addition to individuals aged 40 years and above, children aged 10–15 years living in households with an eligible adult were also included. The survey also collected information on the causes of blindness among other age groups by asking the head of the household if there were any other individuals who were visually impaired and then examining them, irrespective of their age.

### Sample size calculation

Parameters considered in calculating an appropriate sample size were:

○ Assumed prevalence of blindness among those aged 40 years and above (based on previously conducted small surveys) [12-19,21]: 5%

○ Relative precision: 0.5%

○ Confidence limits: 95%

○ Response rate: 85%

○ Design effect: 2

Based on the above, the sample size was calculated to be 15,375 persons aged 40 years or above.

### Sampling Process

A total of 50 individuals aged 40 years and older were enumerated in each cluster. In small villages, if there were less than 50 eligible adults living in the village, the nearest village, which was geographically contiguous, was included and enumerated until the requisite number were identified.

### Enumeration procedures

Proper enumeration is of crucial importance in a cross sectional/prevalence survey, providing the correct denominator for determining blindness and low vision rates.

#### Mapping and Identification of cluster segment for survey

Liaison Officers visited survey villages in advance where they met village elders to explain the purpose and procedures of the survey, to obtain consent for undertaking the survey and to request full participation of all eligible persons. Requests were also made for support in terms of determining the boundaries of the cluster, providing local guides to assist the enumeration team, and identifying a suitable location for the examination site.

If the cluster was large, it was first divided into fairly equal segments thought to be large enough to generate 50 adults. The segments were numbered and the numbers written onto pieces of paper, properly folded to conceal written numbers. A village elder was asked to randomly select one piece of paper to identify the survey segment. If the cluster was small, the whole cluster was included in the enumeration.

Once the cluster segment was selected, the enumeration team identified the centre of that particular segment by going around the circumference of the segment with the village volunteers and then identifying the approximate centre. The enumerators then chose a random direction in which to proceed by spinning a bottle.

#### Enumeration protocol

Enumerators worked in pairs from the random start. Enumeration proceeded systematically wherein an advance team of supervisors first visited households where they interviewed family members to identify households with eligible individuals and allocated a survey house number. The core field investigators went house- to- house completing the enumeration details of each eligible individual after obtaining informed consent. In urban clusters, identification of the streets/blocks was derived from municipal maps provided by the State governments. If a house was locked at the first visit, information was given to the neighbours that the team would return later in the day. Repeat visits were made the same day to gather information about the locked house. If contact could not be established after two visits the household was categorised as a non- responding household.

The process of enumeration was continued until 50 subjects aged 40 years and above had been enrolled. If the last household had more than one eligible individual, all were included even if the total exceeded 50. At the central examination site, age was verified once again against an events calendar (Table 2), and if a person was thought to be below the age of 40 years, he/she was examined but not included in the survey sample.

**Table 2:** Events calendar used to determine age of subjects in 2005

**Event**	**Year**	**Present age if born in this year**
Start of Second World War	1939	66
End of Second World War	1945	60
Queen of England visit to Nigeria	1957	48
Nigerian Independence	1960	45
Assassination of Premier of Northern Nigeria	1966	39
Nigerian Civil War started	1967	38
Change of money to Naira	1972	33
Assassination of General Murtala	1976	29
Change to Shagari civilian government	1979	26
Football World Cup	1994	11

Typically 2–3 days were required for each cluster. When clusters were close to each other, an advanced team of enumerators started work in the next cluster on the 2^nd ^day of the clinical examination so that such cluster could be completed in two days. However, if clusters were far apart, the entire team stayed in the cluster which meant that 3 days were required.

### Ethical Approval

The study adhered to the tenets of the Declaration of Helsinki and was approved by the Ethics Committee of London School of Hygiene and Tropical Medicine and Nigeria's Federal Ministry of Health.

### Informed Consent

Informed consent was obtained from the head of the household and all adult respondents at the time of enumeration. The objectives of the survey and the examination process were explained to those eligible in the local dialect, in the presence of a witness. A subject was examined only after informed consent was obtained. In case of children, consent was obtained from a responsible adult household member.

### Registration and Interview

All enumerated eligible respondents were requested to come to the 'makeshift clinical station' set up in each cluster which was located as close as possible to the cluster residents. Eligible respondents were registered and allocated a unique identification number after verifying their age and residency status. The registration team kept a record of how many enumerated individuals attended the clinical site and this information was given to the enumeration supervisors so they could follow up non-responders. Information on the age, gender, ethnic group, occupational status, religion, educational attainment and household sanitation (sources of water and availability of latrine) were recorded during registration from a responsible adult member of the household.

The interviewer also systematically identified one out of every seven adults that reported to the examination site for a detailed eye examination for collecting normative data (yellow card). The purpose of the normative database was to determine the distribution of ocular variables in normal adult Nigerian eyes (e.g. intraocular pressure, cup disc ratio etc) to give a range of values which could be considered normal for this population.

### Anthropometric measurements and Blood Pressure

Height was recorded to the nearest tenth of a centimetre while weight was recorded to the nearest 100 grams using standard equipment. These measurements were not recorded if a person could not stand erect or was physically disabled. The weighing scale was calibrated every morning and zero error checked before recording the weight of the individual. Blood pressure was recorded using an Omron Wrist Instrument (Omron Healthcare Ltd, Milton Keynes, England). Three readings were taken at least 5 minutes apart after the subject was made comfortable. The instrument was calibrated every morning.

#### Visual acuity measurement

Visual acuity (VA) was measured at the central examination site (in daylight out of direct sunlight) in a shaded area. The ophthalmic nurse recorded whether the participant arrived with distance spectacles, usually wore distance spectacles but had forgotten to bring them, had been prescribed distance spectacles but did not habitually use them, or whether the glasses were broken. Participants were also asked if they used reading glasses. After explanation and demonstration, all participants had the unaided VA of each eye measured at 4 meters using a 'reduced logMAR tumbling E chart' [41-43]. Participants sat for this assessment with the chart 1 meter above the ground. One field worker pointed out the Es in turn while the ophthalmic nurse counted the number correctly identified using a counter. Testing stopped if all 3 letters optotypes on a row were not seen. After measuring the VA in each eye, both eyes were assessed together.

If a participant was unable to read any letters or read only one letter with one or both eyes at 4 meters, the VA was retested at 1 meter. Distance VA was measured in all participants without distance spectacles even if they habitually used them (unaided VA). Those who had distance glasses were reassessed wearing their available glasses (presenting vision). Participants who could not see any letter at 1 meter were assessed by the community ophthalmologist, for finger counting, hand movements and light perception (PL/NPL) in a darkened room. Participants who did not understand the test or who had communication difficulties were assessed and their vision was recorded as ' believed blind' or 'believed not blind'.

The reduced LogMAR 'E' chart was used because of ease of administration and standardization as well as the relative lack of familiarity with the Roman alphabet in Nigeria. The 'E' optotypes on the chart are arranged according to the logMAR scale with three letters per line (total of 30 optotypes), each with a different orientation [42,43]. This chart has been used in other population based surveys [32,33] and allows logMar scores to be converted to Snellen's equivalents.

**Figure 2:**
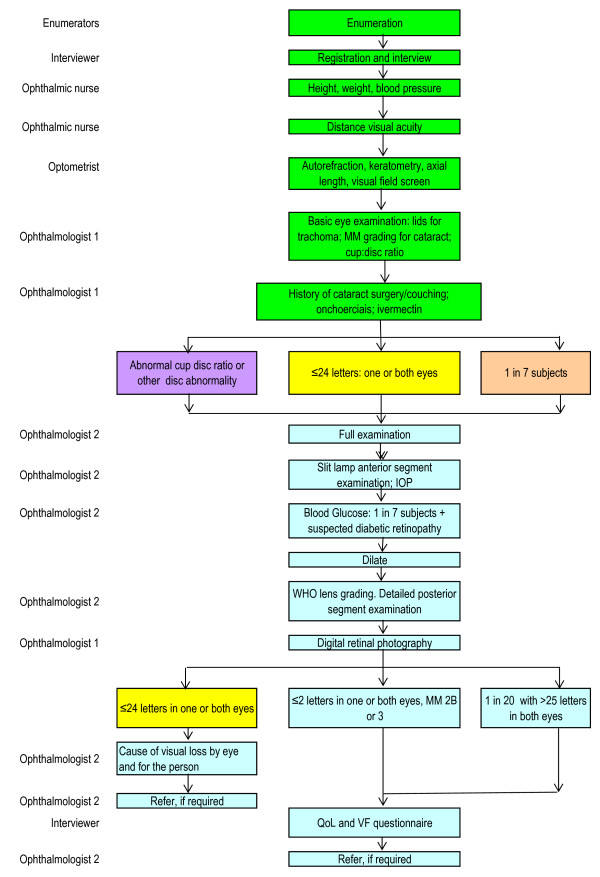
Flow Chart of Examination Protocol.

Participants with LogMar score of ≤ 24 optotypes (i.e. < = 6/12) in one or both eyes were marked as "red cards" while those with a score of 25 or more were marked as "green cards". This division defined the subsequent sequence of examinations that each individual underwent. Participants with red cards had extensive examination including dilated funduscopy. A flow chart of the survey examination procedures is presented in Figure 2.

#### Refracto-keratometry, perimetry and ultrasonography

All participants were then refracted and had their K readings measured using an autorefracto-keratometer (Takagi ARKM-100, Takagi Seiko, Japan) that was regularly calibrated. If automated readings could not be obtained, because of media opacity or lack of cooperation, refraction was done manually by an optometrist. All those with a VA of <6/12 in one or both eyes had their corrected VA measured using the subjective refraction based on autorefraction readings. This was done to estimate the contribution of refractive error to participants' visual impairment.

All adult respondents then had visual field testing using a Humphrey Frequency Doubling Technology (FDT) visual field instrument (Carl Zeiss Meditec AG Jena Germany) set on the N-30 screening mode, after explanation and demonstration. If there were 2 or 3 false positives and/or 2 or more fixation failures testing was repeated after further explanation. Threshold visual field testing was undertaken if there were one or more FDT fields with severe loss, two or more fields with moderate loss or three or more fields with mild loss (in the absence of visual axis opacities) or glaucomatous disc changes i.e. Cup Disc Ratio (CDR) of more than 0.6 or cup asymmetry of greater than 0.2 or notch and/or IOP >20 mm Hg. Anterior chamber depth (ACD), lens thickness and axial lengths were then measured using an ultrasound A-scan (Bioline Biometer OPTIKON 2000 S.p.A Roma Italy) which was regularly calibrated.

#### Basic eye examination

The 'community' ophthalmologists then elicited a history of hypertension, diabetes mellitus, glaucoma, trachoma or ocular trauma in one or both eyes. Participants were asked if they had skin changes typical of Onchocerciasis by showing them pictures of nodules and "leopard skin". In areas endemic for Onchocerciasis participants were asked if they had taken Mectizan in the previous12 months. Each participant had an anterior segment examination using a torch to elicit signs of trachoma, pterygium, conjunctival disease and corneal pathology. Lenses were graded using the Mehra – Minassian system [44]. Individuals with evidence of surgery (eyelid, cataract, glaucoma etc) were noted. Those who had had cataract surgery or couching were asked where the surgery was performed, what type of surgery was undertaken and whether they used aphakic correction (following non-IOL surgery or couching). Posterior segments were examined through an undilated pupils with direct ophthalmoscope to assess vertical cup:disc ratios (CDR), CDR asymmetry between the two eyes (defined as CDR asymmetry >0.2), and the presence of splinter hemorrhages on the optic disc. Other retinal pathology was also recorded e.g. vascular retinopathy, retinitis pigmentosa, age related macular degeneration.

All participants suspected to have diabetic retinopathy and those selected for the normative database (i.e. the 1 in 7 "yellow cards") had a random blood sugar tested using one-touch blood sugar machine (OMRON one touch ultra blood glucose meter).

#### Detailed eye examination

Three categories of individuals proceeded to a more detailed examination by the clinical ophthalmologists i.e.:

1) Those with a presenting VA of < = 6/12 in one or both eyes (red cards)

2) One in seven participants (yellow cards) for the normative data base and

3) Subjects aged 40 years and more, with a CDR of >0.6 or CDR asymmetry of >0.2 or who had splinter hemorrhages on the disc, irrespective of their visual acuity.

The 'clinical' ophthalmologist assessed iris color, pupillary light reflexes and anterior chamber depth using a slit lamp microscope (Zeiss SL 115 Classic Slit Lamp, Carl Zeiss Meditec AG Jena Germany) and Von Herrick's method. Intraocular pressures were measured in each eye using a Goldmann applanation tonometer. Persons with intraocular pressure above 20 mmHg, CDR >0.6, CDR asymmetry of >0.2 between the two eyes and Von Herrick's grade of <3 had gonioscopy using a gonio lens without flanges (Volk 2 Mirror Lens with no flange).

All participants undergoing detailed examination, apart from those with narrow angles, had their pupils dilated with 1% tropicamide and/or phenylephrine 10%. Anterior and posterior segments were examined at the slit lamp using an 81D Aspheric condensing lens (Volk) and bilateral indirect ophthalmoscope. Lens grading was performed using the WHO grading system [45]. Vertical CDR and CDR asymmetry were reassessed. The posterior segment was examined for optic nerve disc notching, splinter hemorrhages and retinal pathology. Age related macular degeneration (ARM) and its classification into dry and wet ARM.

For all participants with a presenting VA of < = 6/12 in one or both eyes, the clinical ophthalmologist selected all the disorders that may have contributed to visual impairment for each eye, from a list of disorders. Cataract and refractive error were identified as the cause of visual impairment using the following definitions: significant cataract defined as grade 2B or 3 (MM grading) and/or a score of 2 or 3 for any WHO lens grading; significant refractive error and uncorrected aphakia were defined as an acuity of <6/18 before refraction which improved to ≥ 6/18 after refraction. If there was more than one disorder for an eye then one disorder was selected for that eye using the following decision tree:

• If one disorder led to the other then the primary disorder was chosen

• If one disorder was judged to be a more significant cause of visual loss than other disorders then most significant disorder was selected

• If however all disorders were judged to contribute equally to visual loss then the disorder more amenable to treatment, or to prevention, was chosen.

In addition to describing the anatomical disorder responsible for visual loss in each eye the underlying cause was determined for each eye e.g. age related, surgical complications, trachoma, congenital etc.

Next, the principal disorder responsible for visual loss for the person was decided. If the main disorder differed between the two eyes the disorder for the person was determined using the following criteria:

• The disorder most amenable to treatment was selected.

• If this did not apply, then the condition most amenable to prevention was selected.

• If this did not apply, then the disorder responsible for visual loss in the better seeing eye was selected.

The underlying cause for principal disorder was the underlying cause for the person.

All adult participants with visual impairment in one or both eyes (red card participants) had a digital fundus images taken using Carl Zeiss digital fundus camera (Zeiss VISUCAM Lite Desk Top Fundus Camera, Carl Zeiss Meditec AG Jena Germany). In capturing the images the camera was focused on to the posterior pole covering the optic nerve head and the macular region through a dilated pupil.

### Barriers to Uptake of Eye Care Services

The following participants were asked why they had not attended eye clinic/surgery:

• Those with a presenting VA of <6/60 in one or both eyes (i.e. score of <02);

• Those with cataract MM grades of 2B or 3 in one or both eyes and

• Those with trichiasis in one or both eyes.

Four reasons were recorded on the form with categorization into first, second, third and fourth according to the participant's response. The options on the form were ascertained during previous studies conducted in similar settings [13,16,46]. The options included cost, no time, no need, fear of surgery etc. The instrument adopted a semi-open ended format so that information on other barriers could also be documented.

### Quality of life questionnaire schedule

A quality of life instrument was administered to all respondents who had a visual acuity of <6/60 in one or both eyes, or who had MM cataract grade of '2B' or '3' in one or both eyes. The questionnaire was also administered to a randomly selected sample 1 in 20 participants who were not visually impaired (green cards). These questionnaires were adopted from instruments used in earlier surveys in developing countries [47,48]. The standardized quality of life instrument, which was pre-tested in Nigeria during a pilot study, had been used in the national blindness surveys in Bangladesh and Pakistan [32,33]. The English prototype was translated into the major local languages and accuracy of the translation was tested by back translation into English. The two translated English versions were compared and changes made, if necessary. Translation and back-translation was undertaken by different people to ensure reliability.

A 4 point rating scale was used for a series of questions related to difficulties in relation to activities for daily living (ADL), face recognition, recognition of small items, dark adaptation, colour recognition, reaching for food on a plate or a glass of water, self care activities (e.g. bathing, personal grooming), activities related to mobility, social activities like participation in social events and mental perception of the visually impaired adults. They were also queried on whether they needed help in carrying out self care activities.

### Survey Personnel

The survey was conducted by a team of highly skilled and committed professionals from Nigeria with technical support from personnel at ICEH, London and the National Eye Centre, Kaduna, Nigeria. Technical support from ICEH consisted of an ophthalmologist, epidemiologist, biostatistician and a technical officer experienced in instrument maintenance. A Project Advisory Committee (PAC) was formed to guide the survey. Members of the PAC included Federal Ministry of Health officials, NPPB coordinator, international NGOs based in Nigeria, leading ophthalmologists and academics, the survey team coordinator and ICEH staff. The PAC met periodically to review progress and solve outstanding problems. The SSI Nigeria Country Office provided all the logistic and administrative support for the survey.

The core team for the survey consisted of the Project Coordinator and the Financial Advisor, two clinical ophthalmologists, two community ophthalmologists and two optometrists (one of each cadre for the two field teams). Most members of the core team remained constant throughout the survey to ensure uniformity in data collection across the country.

Other members of the team were recruited for each GPZ. This was necessary because all 'front line personnel' needed to be fluent in the local languages and familiar with local cultural practices. The following were recruited to each team for each GPZ: 1 liaison officer, 2 ophthalmic nurses and 6 enumerators, most of whom were ophthalmic nurses. One enumerator acted as the field supervisor, another was trained in interviewing while another examined non-study participants with eye complaints. Each team also had a cook and 2 drivers who remained constant throughout the survey. The drivers also helped the field teams as and when required. A total of 140 Nigerian field staff took part in the survey across the country.

### Timeline

Survey planning started in April 2004 and a consensus meeting was held in Nigeria which was attended by the Federal Government of Nigeria, senior ophthalmologists experienced in population based research and international NGOs. During this meeting the first draft of the protocol was written which was then extensively reviewed by technical experts. Procurement of equipment and recruitment of staff was completed and training of the team for the first phase of the survey was undertaken in January 2005. Training was followed by a pilot survey in two clusters in Kaduna State.

Data collection was split into 6 phases with one GPZ being surveyed in each phase. There was a gap of a few weeks to a few months between each phase to avoid the rainy season when field work was impossible (June to August) and which allowed survey team members to visit their families.

Data were collected by the two clinical teams who worked in two different locations concurrently. Each clinical team was supported by a dedicated enumeration team.

#### Training and quality control measures

An ophthalmologist and epidemiologist from the International Centre for Eye Health, London (ICEH), and a technician, all experienced in population based surveys, trained the 4 ophthalmologists and 2 optometrists (the core team) at the onset of the survey for 4 weeks, in sampling procedures, enumeration processes, use and handling of equipment, examination procedures and recording data. Further 2 week periods of training took place in each GPZ to retrain the ophthalmologists and optometrists, and to train the local, zonal staff recruited from the GPZ i.e. ophthalmic nurses, enumerators, interviewers and liaison officers. The liaison officer was trained in cluster identification, informing the relevant communities and arranging the examination sites. The enumerators and interviewers were trained in enumeration procedures, mobilization and registration of participants, while the ophthalmic nurses were trained in VA measurement and recording.

During zonal training sessions, inter-observer agreement (IOA) assessments were conducted for MM grading and ascertainment of causes of visual impairment among the ophthalmologists, while the IOA assessments for VA were conducted for the ophthalmic nurses. A skilled Optometrist was the 'gold standard' for recording VA. For all these personnel, intra-class correlation coefficient and Kappa were calculated and reasons for differences were identified and discussed so that a consensus could be achieved according to the operational definitions. Ophthalmic nurses with low Kappa scores compared to the 'gold standard' in the VA measurement were replaced with ophthalmic nurses who had good Kappa values. The zonal training sessions were supervised by an epidemiologist and ophthalmologist. A pilot study was conducted after each zonal training session.

A Survey Manual (Standard Operating Procedures) describing all the steps involved in the survey and the description of each procedure was prepared and given to all team members. This was regularly used during training (where each individual team member was required to read the manual) and for reference during field work.

Other quality assurance procedures included:

- Regular monitoring of the ophthalmic nurses and the optometrists was done by the Community Ophthalmologists who were designated as team leaders to help with administrative issues in the field.

- Periodic monitoring visits were made by the Project Coordinator to both teams where he monitored practices and took corrective action as and when required.

- Monitoring of the survey was also undertaken by officials from the NPPB.

- The technical team from ICEH and technical experts from the Region also regularly visited the teams in the field and observed the work being undertaken by both the enumeration and clinical eye examination personnel.

- A customized data entry package was developed and used where range and consistency checks were built in to facilitate data entry.

- Double data checking was used to improve the quality of data entry. This meant that one clerk entered the data for one cluster and then passed on the data to a companion for a 100% verification of the data by comparing with the data forms for consistency.

- Data cleaning programmes were written in STATA 10.0 (Stata Corp, and all data were cleaned using the programs. This was supported by a random verification of the data entered with the data recorded on the forms.

#### Non responders for participation in the survey

Participants who were enumerated and eligible for the survey but who did not attend the examination site were visited at home to determine the reason for their absence and to convince them to attend. If they were unwilling to attend the examination site but were willing to be examined at home, the community ophthalmologist conducted a basic eye examination, including VA measurement and refraction using a NIDEK portable Auto Ref/Keratometer (Model ARK-30, Nidex Co. Ltd, Gamagori Aichi, Japan).

Individuals who refused to participate, or who were not available for the 2 days the team was in the cluster, were categorized as non responders and information was obtained on their visual status (either believed not blind or believed blind) by observation or from information provided by relatives or neighbors.

**Figure 3:**
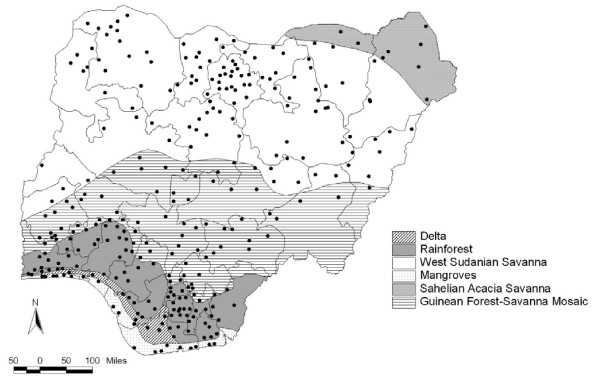
Ecological zones and clusters covered in the survey.

A total of 310 clusters were identified for the survey spread across all the ecological zones in the country (Figure 3). The survey had to be abandoned in 3 clusters in the South South GPZ due to civil unrest and in 2 clusters in the South West GPZ as communities refused to participate. The survey proceeded smoothly in the rest of the country. A total of 305 clusters could therefore be covered among the 310 initially identified (98.4%).

#### Data management

A record sheet was completed for each eligible enumerated participant, after being cross-checked for errors by the community ophthalmologists in the field and the project coordinator in the office. The data were subsequently entered into a customized database (with built in range and consistency checks) by an experienced data officer and independently crosschecked by a second data officer. Data cleaning and analysis is being done using STATA 10.0 (StataCorp LP, Texas, USA) by a dedicated statistician at ICEH.

The Visual fields, autorefractokeratometers readings, and A-scan biometry readings were recorded, printed and attached to the record forms. Fundus images were stored on hard drives of the fundus camera and written on CD plates for reading and grading at the grading center Moorfields eye hospital London. All images will first be examined for quality and categorized as excellent, good, fair, borderline and ungradeable. For images where the quality is good or excellent, the final diagnosis will be based on the images while in case of fair/borderline images, two clinicians will review the findings to establish the diagnosis. If the images are ungradeable the clinical diagnosis will be used. For gradable images the retina and optic disc will be reviewed, and a diagnosis made based on the appearance of the image e.g. diabetic retinopathy, toxoplasmosis, onchocerciasis, age related macular degeneration, myopic fundus, glaucoma, optic atrophy or other retinal pathology. Quality assurance will be ensured by a senior grader (who has experience of retinal findings in onchocerciasis) who will verify a random 10% of images that are graded as normal as well as abnormal.

The FDT visual fields will be read by an independent, experienced examiner who will grade them as normal; blind (i.e. central field of <10^0^); severely visually impaired (i.e. central field of 10 to <20^0^); or other field loss.

#### Service component

All participants with visual impairment were referred to the nearest eye facility. People with operable cataract were referred to the cataract service centers where free or subsided cataract surgery had been organized for survey participants. A total of 3,620 people had cataract surgery as a direct result of taking part in the survey, and 5,800 pairs of reading glasses and over 200 pairs of aphakic glasses were distributed at no cost. Participants with mild ocular or systemic complaints were also treated as were over 30,000 non-survey participants who attended the examination sites with ocular complaints.

The survey was owned by the Federal Government of Nigeria and supported by the respective State governments and therefore will serve as a baseline to be used in planning for eye care services in Nigeria. A survey of this magnitude could not have been successfully implemented without excellent support from the federal government and the local administration at State and LGA level. This partnership was crucial to the success of the survey.

## Abbreviations

ACD: Anterior Chamber Depth; ADL: Activities for Daily Living; ARKM: Auto refracto kerato meter; ARM: Age related macular degeneration; CD: Compressed Disc; CDR: Cup Disc Ratio; FCT: Federal Capital Territory; FDT: Frequency Doubling Technology; GDP: Gross Domestic Product; GPZ: Geo political zones; ICEH: International Centre for Eye Health; IOA: Inter Observer Agreement; IOL: Intra Ocular lens; LGA: Local Government Authority; MM: Mehra-Minassian; NPL: No perception of light; NPPB: Nigeria National Program for Prevention of Blindness; PAC: Project Advisory Committee; PL: Perception of light; PPP – Purchasing Power parity; PPS: Probability proportional to size; SSI: Sight Savers International; VA: Visual Acuity; WHO: World Health Organization

## Competing interests

None of the authors have any competing interests (financial or otherwise) in the publication of the results which is a true reflection of the state of eye health in Nigeria. The study was funded by a number of International Non Governmental Organizations including Sight Savers International and one of the authors (EH) is the Country Director of Sight Savers International in Nigeria.

## Authors' contributions

DB: Conceptualization and formulation of study protocol; Training; Reading and revising manuscript. GCE: Conceptualization and formulation of study protocol; Training; Monitoring of Study Implementation; Reading and revising manuscript. RMM: Finalization of study protocol; Coordination of Study; Monitoring of Study Implementation; Reading and revising manuscript. KF: Contributed to Study Design; Acquisition of data; Reading and revising manuscript. AMM: Contributed to Study Design; Acquisition of data; Reading and revising manuscript. TA: Contributed to Study Design; Acquisition of data; Reading and revising manuscript. EC: Contributed to Study Design; Acquisition of data; Reading and revising manuscript. EG: Contributed to Study Design; Acquisition of data; Reading and revising manuscript. EE: Monitoring of Study Implementation; Reading and revising manuscript. AA: Finalization of study protocol; Reading and revising manuscript. FH: Finalization of study protocol; Monitoring of Study Implementation; Training; Reading and revising manuscript. JJ: Finalization of study protocol; Reading and revising manuscript. OC: Finalization of study protocol; Reading and revising manuscript. PSL: Contributed to study design; Training; Monitoring and quality assurance; Reading manuscript. MGVS: Contributed to Study Design; Training; Monitoring of Study Implementation; Quality assurance of data; Data cleaning; Data Analysis; Drafting and revising manuscript and given final approval for the version to be published

## Pre-publication history

The pre-publication history for this paper can be accessed here:


